# A Combination of Two Rare Coronary Anomalies Makes It Even Rarer: Right Sided Single Coronary Artery with Dual Left Anterior Descending Artery

**DOI:** 10.1155/2016/4905941

**Published:** 2016-05-18

**Authors:** Aram Barbaryan, Theodore Addai, Monahar Kola, Muhammad Wajih Raqeem, Sergey Barsamyan, Aibek E. Mirrakhimov

**Affiliations:** ^1^Department of Medicine, HSHS Saint Mary's Hospital, Decatur, IL 62521, USA; ^2^Department of Cardiology, HSHS Saint Mary's Hospital, Decatur, IL 62521, USA; ^3^Oxford Heart Rhythm Service, John Radcliffe Hospital, Oxford University Hospitals NHS Foundation Trust, Headley Way, Headington, Oxford OX3 9DU, UK; ^4^Department of Medicine, University of Kentucky, Lexington, KY 40506, USA

## Abstract

An 82-year-old female with history of hyperlipidemia and hypertension presented to the clinic with chief complaint of nonradiating chest tightness accompanied by exertional dyspnea. Cardiac catheterization showed the absence of left coronary system; the entire coronary system originated from the right aortic sinus as a common trunk which then gave off the right coronary artery and the left main coronary artery. Cardiac catheterization demonstrated also another rare coronary anomaly: dual left anterior descending artery. Patient underwent percutaneous coronary intervention and subsequent multidetector computed tomography angiography confirmed the above angiography findings. Patient was subsequently discharged home on double antiplatelet therapy with aspirin and clopidogrel and has been asymptomatic since then.

## 1. Introduction

In a single coronary artery (SCA) the entire coronary tree arises as a single trunk from ascending aorta and no evidence of second coronary artery is found [1]. The prevalence of SCA in angiographic series fluctuates between 0.014 and 0.066 percent [2, 3]. In 43 percent of cases SCA is associated with other major congenital heart anomalies [1]. Dual left anterior descending (LAD) artery was first described by Spindola-Franco et al. in 1983. Based on conventional coronary angiography and CT angiography data the prevalence of dual LAD is estimated to be 1% and 4%, respectively [4, 5].

## 2. Case Presentation

An 82-year-old female with history of hyperlipidemia and hypertension presented to the clinic with chief complaint of nonradiating chest tightness with exertion which started several months before this presentation but got significantly worse in the last one month. Patient had normal vital signs and unremarkable physical examination. The patient's home medications were simvastatin, metoprolol, isosorbide mononitrate, and aspirin. The patient had a stress radionuclide myocardial perfusion imaging study five months prior to the presentation results which did not show inducible ischemia. 12-lead electrocardiogram (ECG) showed T wave inversions in lateral precordial leads that were unchanged from ECG five months earlier. Cardiac biomarkers were within normal limits. Transthoracic echocardiogram showed ejection fraction of 60%, and no valvular abnormalities were found. Decision was made to perform elective cardiac catheterization.

**Figure 1:**
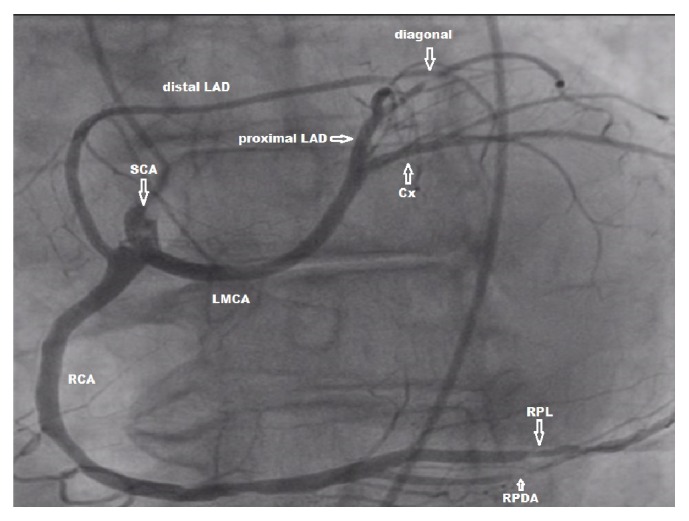
Coronary angiographic image (left anterior oblique 30°/0° projection, field of view 25 cm^2^) showing single coronary artery (SCA) arising from the right sinus of Valsalva as short common trunk which divides into right coronary artery (RCA) and left main coronary artery (LMCA). LMCA further bifurcates into the proximal left anterior descending (LAD) artery and the circumflex (Cx) artery. The distal LAD originates separately from the right coronary artery. Stenotic lesions in right posterolateral (RPL) and right posterior descending (RPD) arteries are also depicted.

A 5-French sheath was placed over the guidewire into the right femoral artery. The left coronary system was not present; the entire coronary system originated from the right aortic sinus as a common trunk which then gave off the right coronary artery (RCA) and the left main coronary artery (LMCA) (Figure 1). The common trunk was cannulated with a 5-French right Judkins catheter tip #4. Distal LAD was found to have 95% stenosis in its midportion. A significant lesion in diagonal artery up to 95% was found with fair distal antegrade flow. RCA was dominant compared to the left system and appeared to have two lesions, one in the posterior descending artery (PDA) branch up to 90% and another one in posterolateral branch up to 80%. Left ventricular (LV) angiography showed good LV contractility. Subsequently four drug eluting stents (DES) were placed in diagonal artery (2.25 × 18 mm), distal LAD (2.25 × 12 mm), PDA (3.0 × 23 mm), and posterolateral branch of RCA (2.25 × 18 mm) with excellent TIMI 3 flow.

**Figure 2:**
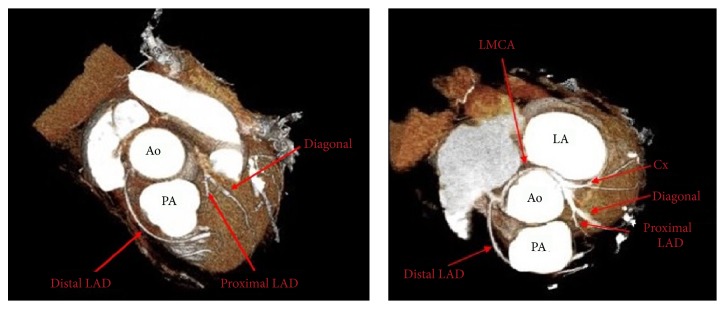
Colored three-dimensional (3D) volume rendered CT angiography images showing single coronary artery (SCA) arising as a common trunk from the right sinus of Valsalva and bifurcating into the right coronary artery (RCA) and the left main coronary artery (LMCA). RCA gives rise also to distal LAD which courses anterior to the PA on its way to the left ventricle. LMCA crosses the base of the heart turning posteriorly behind the aorta dividing into proximal left anterior descending artery (LAD) and circumflex artery. Left atrium (LA), aorta (Ao), and pulmonary artery (PA).

64-row multidetector computed tomography (MDCT) scanner (Siemens SOMATOM Definition AS 64 slice scanner, Germany) was used to further characterize the course of anomalous coronary artery. Images were acquired in the craniocaudal direction with 0.6 mm slice thickness. It confirmed cardiac catheterization findings showing entire coronary system originating from the right aortic sinus (Figure 2). The LMCA coursed in a retroaortic fashion between the aorta and the left atrium on its way to the left ventricle. The LMCA bifurcated into the proximal and mid-LAD artery as well as the Cx. Only the proximal and mid-LAD originated from LMCA. The distal LAD originated separately from the right coronary artery and then coursed anterior to the pulmonary artery on its way to the left ventricle supplying the distal portion of the anterior intraventricular groove.

Patient was subsequently discharged home on double antiplatelet therapy with aspirin and clopidogrel and has been asymptomatic since then.

## 3. Discussion

This type of SCA is classified as RII-P according to Lipton classification. In this type of SCA the entire coronary tree arises as a common trunk from the right coronary sinus dividing into right coronary artery (RCA) which has a normal course and left main coronary artery (LMCA). Then LMCA crosses the base of the heart turning posteriorly behind the aorta dividing into LAD and Cx arteries [6]. In the malignant or interarterial type of this anomaly when LMCA crosses between aorta and pulmonary artery patients might present with sudden death due to compression and kinking of LMCA especially during physical exertion [2, 7, 8]. Otherwise the posterior variant (this case) does not carry increased cardiovascular risk in the absence of atherosclerotic coronary artery disease (CAD) and other congenital cardiac abnormalities [1, 7, 9]. However 15% of SCA patients have myocardial ischemia in the absence of atherosclerotic CAD which might be a direct result of abnormal coronary anatomy [10]. Because of its rarity there are currently no guidelines on the management of patients with SCA. Revascularization is indicated in cases of atherosclerotic CAD and ischemia [11]. Invasive management strategies in patients with SCA and atherosclerotic CAD are very rare and pose high risk since cannulation of common trunk by large catheter might be poorly tolerated given the fact that the whole heart gets its supply from that common single trunk [12].

**Figure 3:**
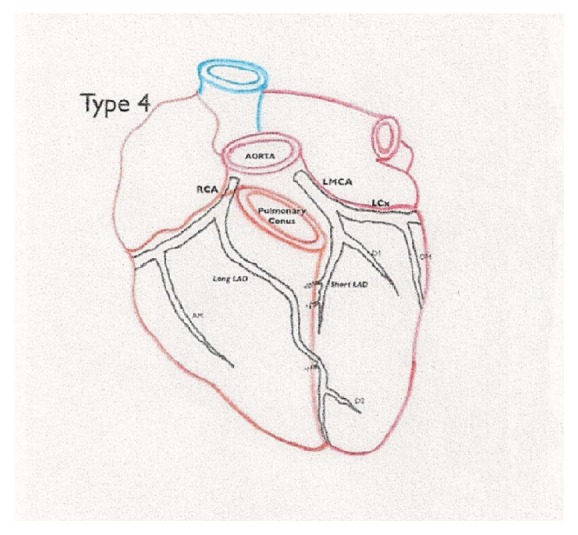
Type 4 dual LAD configuration by Spindola-Franco et al. RCA: right coronary artery; LMCA: left main coronary artery; LAD: left anterior descending artery; LCx: left circumflex artery. Reprinted with permission from [5].

The uniqueness of our case is the combination of SCA with another rare coronary anomaly: dual LAD. Dual LAD is defined as bifurcation of anterior descending artery into a short LAD terminating in proximal anterior interventricular sulcus (AIVS) and the long LAD that has variable course returning to AIVS distally. Short LAD supplies the anterior interventricular septum and the long LAD supplies anterolateral wall and the apex. So far 9 subtypes of dual LAD have been described by different authors. Our case represents type IV of dual LAD where long LAD arises from RCA lying on the anterior surface of the right ventricle and sharply turning to descend into the AIVS (Figure 3). Recognition of anatomic variants of dual LAD is crucial for correct identification of these vessels during surgery and coronary interventions for coronary artery disease [4, 5].
